# Physiology-Driven Irrigation Scheduling in *Ananas comosus* via Hybrid Machine Learning: UAV-Based Phenotyping of Water-Related Traits Coupled with FAO-56 Soil Water Balance

**DOI:** 10.3390/plants15142112

**Published:** 2026-07-08

**Authors:** Jorge Enrique Chaparro, Jose Edinson Aedo, Nelson Barrera Lombana

**Affiliations:** 1 Research Group TICTROPICO, International University of the American Tropics, Unitrópico, Yopal 850001, Casanare, Colombia; 2 Research Group SISTEMIC, Faculty of Engineering, University of Antioquia, UdeA, Medellín 050023, Antioquia, Colombia; jose.aedo@udea.edu.co; 3 INFELCOM Research Group, Universidad Pedagógica y Tecnológica de Colombia, UPTC, Sogamoso 152210, Boyacá, Colombia; nelson.barrera@uptc.edu.co

**Keywords:** UAV multispectral imagery, IoT sensor networks, physics-informed machine learning, precision irrigation, soil moisture estimation, FAO-56 Penman–Monteith, *Ananas comosus*, canopy phenotyping

## Abstract

Field-based phenotyping of water-related traits for precision irrigation in tropical agroecosystems poses a persistent methodological challenge, driven by high climatic variability and the complex water-use physiology of Crassulacean Acid Metabolism (CAM) crops such as pineapple (*Ananas comosus* var. MD2). We developed and validated a Physics-Informed Machine Learning (PIML) framework that integrates high-resolution UAV multispectral imagery, IoT-based microclimatic records, and a mechanistic soil water balance based on the FAO-56 Penman–Monteith standard to predict plot-scale soil moisture depletion as a proxy of plant water status. A six-month field campaign (March–August 2022) across 25 georeferenced commercial pineapple plots in the Colombian Orinoquia piedmont yielded a spatiotemporally balanced dataset of N=150 observations. Soil-adjusted vegetation indices (OSAVI, MSAVI) outperformed standard NDVI for capturing water-related canopy traits, effectively decoupling spectral responses from substrate noise. A Gradient Boosting regressor achieved $R^{2}=0.842$ and RMSE=0.0705 on a normalized target scale, corresponding to a 7.05% error over the prediction range, while the traffic-light Decision Support System (DSS) for irrigation scheduling reached 91.1% accuracy (Cohen’s Kappa =0.91). Incorporating daily soil moisture depletion as a mechanistic feature improved predictive accuracy over a spectral-only baseline ($\Delta R^{2}=+0.052$) and anchored predictions within a physically consistent framework based on the FAO-56 water balance, with no false negatives observed for water deficit detection in the hold-out validation set. This framework advances high-throughput, population-scale phenotyping of water-related traits in open-canopy CAM crops, establishing a transferable methodology for operational precision irrigation under tropical savanna conditions.

## 1. Introduction

Monitoring crop water status in field conditions is particularly demanding for CAM species, where diurnal stomatal closure decouples canopy reflectance from actual tissue water content during conventional daylight acquisition windows, systematically compromising the interpretation of remote sensing signals [1,2]. Agriculture accounts for approximately 70% of global freshwater withdrawals [3]; the precision with which irrigation is managed therefore determines both resource efficiency and the quality of phenotypic inferences derived from spectral observations. Climate variability in tropical agroecosystems has intensified these pressures, driving the adoption of precision agriculture approaches that rely on predictive, model-based systems [4]. In Colombia, however, implementation remains constrained by limited high-resolution meteorological infrastructure and insufficient research on specialized tropical crops [5].

Pineapple (*Ananas comosus* var. MD2) concentrates these monitoring difficulties. The crop anchors the agricultural economy of Casanare Department, where approximately 15,000 ha of commercial production (as of 2023) sustain smallholder livelihoods [6]. Optimal development requires temperatures of 20–36 °C and annual rainfall of 1500–3500 mm; erratic precipitation and thermal stress reduce fruit caliber and promote root-pathogen pressure [6]. CAM physiology confers nocturnal CO_2_ fixation and intrinsic drought tolerance [2], but prolonged soil water deficit induces parenchyma dehydration while simultaneously suppressing the daytime spectral indicators used to detect early stress onset. This metabolic constraint strongly hampers the use of conventional remote sensing phenotyping pipelines designed for C3/C4 crops, which rely on daytime canopy reflectance to detect early stress [1]. Under these conditions, monitoring hydric status extends beyond irrigation scheduling to serve as a critical phenotypic proxy for plant vigor, biomass accumulation, and water-use efficiency under adverse tropical climates [2,6].

Resolving this incompatibility requires multimodal, dynamic monitoring architectures. Wireless sensor networks with IoT connectivity continuously track soil–plant–atmosphere interactions [7], and when coupled with machine learning, enable predictive estimation of water-related stress at operationally relevant scales. LSTM-RNN architectures and multivariate ensemble models have demonstrated consistent promise for water stress prediction across multiple crop systems [8,9]. Within-field spatial heterogeneity, however, demands centimeter-scale resolution to resolve individual plant variability—a requirement that satellite platforms cannot satisfy. UAV systems equipped with multispectral sensors achieve subcentimeter ground sampling distances, capturing canopy variability that remains invisible at coarser resolutions [10,11].

Despite these advances, a key methodological gap remains in many UAV-based phenotyping pipelines: the widespread reliance on NDVI as a single phenotypic descriptor. In open-canopy crops like pineapple, NDVI is confounded by background soil reflectance, reducing its sensitivity to canopy physiological status [12]. Soil-adjusted indices offer a potential solution: OSAVI and MSAVI partially decouple the canopy physiological signal from substrate reflectance and have shown superior performance to NDVI in sparse or open canopies [13,14]. A second, compounding gap concerns the atmospheric dimension: crop spectral response is jointly modulated by edaphic water availability and vapor pressure deficit, and unimodal spectral approaches cannot disentangle these drivers [15]. While Radiative Transfer Models (RTMs) provide a physical basis for leaf–canopy interactions, their parameterization in heterogeneous open-canopy architectures is computationally prohibitive for operational field-scale deployment. Machine learning offers a flexible alternative by learning non-linear mappings between multimodal spectral signals and physiological states [4,16]. Recent approaches increasingly rely on multimodal sensor fusion, integrating biological response (UAV spectral indices), environmental demand (Reference Evapotranspiration, $ET_{o}$), and ground-truth soil moisture sensors [16]; however, no study has incorporated FAO-56 water balance variables as mechanistic predictors within this sensor architecture for phenotyping in CAM bromeliad systems.

This study develops, to the best of current literature, one of the first physics-informed machine learning framework that couples a FAO-56 Penman–Monteith soil water balance with UAV-based multispectral phenotyping and IoT microclimatic sensing for water-related trait estimation in a commercial CAM crop. We develop and validate a Physics-Informed Machine Learning (PIML) framework that integrates high-resolution UAV multispectral imagery, IoT-based microclimatic records, and a mechanistic FAO-56 Penman–Monteith soil water balance within a Gradient Boosting architecture for soil moisture status phenotyping in *A. comosus* var. MD2. The framework was evaluated over a six-month field campaign (March–August 2022) integrating monthly UAV multispectral flights (MicaSense RedEdge-M) with continuous IoT monitoring across 25 georeferenced plots in the Colombian Orinoquia piedmont. Embedding daily soil moisture depletion ($SM_{\mathrm{Depl}}$) as a physically-derived variable provides hydrological context to the FAO-56 water balance framework and provides the atmospheric context required to interpret CAM-specific spectral responses.

Three objectives structured the investigation: (i) to assess whether soil-adjusted indices (OSAVI, MSAVI) reduce soil background interference relative to standard NDVI as physiological phenotypic descriptors in open-canopy pineapple architectures; (ii) to quantify the predictive improvement conferred by incorporating $ET_{o}$ and $SM_{\mathrm{Depl}}$ as physics-informed features relative to a spectral-only baseline model; and (iii) to validate an operational, phenotyping-based traffic-light Decision Support System (DSS) that classifies soil moisture status into three management zones (Stress, Optimum, Saturation), with priority weighting toward minimizing false negatives in water deficit detection, appropriate for autonomous irrigation triggering in commercial tropical cultivation.

## 2. Materials and Methods

### 2.1. Experimental Design

Five independent commercial plots (1 ha each, total area = 5 ha) were selected for a field-based phenotyping experiment targeting water-related traits in *Ananas comosus*. A stratified random sampling strategy captured spatial heterogeneity in piedmont soil and moisture gradients. Twenty-five permanent sampling units were georeferenced across the plots, at a density of five units per hectare.

Each sampling unit consisted of 10 pineapple plants (*Ananas comosus* var. MD2) in a 2×5 row arrangement. Units were demarcated with four physical stakes and a central white flag (1.5 m height), enabling precise spatial synchronization between UAV spectral data and ground measurements across all orthomosaics. Alphanumeric codes (e.g., B1, T3) maintained traceability across the six-month monitoring period.

We integrated edaphic, spectral, and atmospheric variables into a synchronized spatiotemporal matrix for multimodal data fusion, defining the phenotyping feature space (Table 1).

IoT microclimatic variables were recorded automatically at 10-min intervals throughout the six-month campaign. For multimodal fusion, each UAV acquisition was linked to the mean IoT measurements recorded within the corresponding daytime flight window (10:00–14:00 h) on the same acquisition day. FAO-56-derived variables (Depl, Deficit) represent the cumulative daily values computed for that day from the continuous soil water balance simulated in CROPWAT 8.0. The final dataset comprised N=150 independent observations, generated by the intersection of the 25 spatial sampling units and 6 consecutive monthly acquisition campaigns (25units×6months). This structure enabled machine learning models to capture both temporal phenological dynamics and spatial water availability gradients within the commercial production system at plot scale. The resulting observation-to-predictor ratio (N=150, 34 input features, ratio ≈4.4) is acknowledged as a dimensional constraint; L1/L2 regularization and stratified 5-fold spatial cross-validation were applied specifically to mitigate overfitting risk, consistent with established practice for high-dimensional phenotyping datasets of comparable scale [10,16].

**Figure 1:**
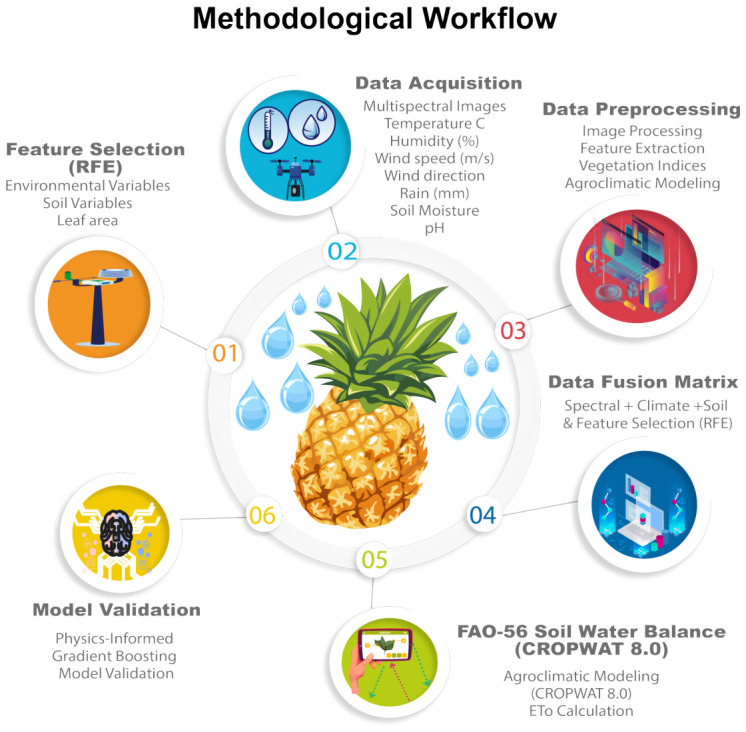
Methodological workflow of the study. The framework integrates multimodal data acquisition (UAV, IoT, manual), data pre-processing, and machine learning modeling for soil moisture status phenotyping.

Multispectral data were acquired using a MicaSense RedEdge-M camera (MicaSense, Seattle, WA, USA) integrated into a DJI Phantom 4 Pro UAV (SZ DJI Technology Co., Ltd., Shenzhen, China). Simultaneously, a custom-built IoT agroclimatic station recorded microclimatic and edaphic variables. Manual measurements at the 25 sampling points, synchronized with UAV flights, provided spectral validation ground truth. The overall methodological workflow is illustrated in Figure 1.

### 2.2. Study Area Description

The experiment was conducted during the vegetative growth phase of a commercially grown pineapple crop (*Ananas comosus* var. MD2) in Tauramena, Casanare, Colombia ($4^{\circ }59^{\prime }$ N, $72^{\circ }45^{\prime }$ W). Tauramena accounts for approximately 42% of Casanare’s pineapple cultivation area, supported by nearly two decades of commercial production. The region exhibits a tropical savanna climate (Aw) with a unimodal precipitation pattern [17].

The site occupies a piedmont zone with high natural drainage capacity, providing favorable edaphic conditions for root health. The monitoring period spanned March to August 2022, covering critical phenological transitions from vegetative development to flowering induction. Given the combination of high atmospheric demand and well-drained soils characteristic of this piedmont setting, plant water status constitutes a key phenotypic trait for yield stability in commercial pineapple production [2,6].

**Figure 2:**
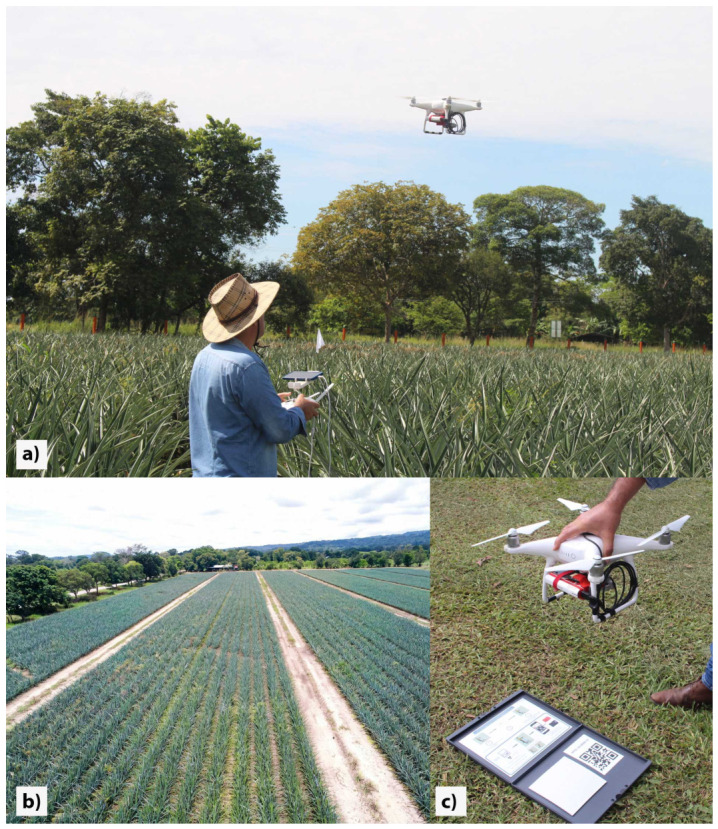
Field experimental setup. (**a**) Aerial close-up of a georeferenced sampling unit marked with a white flag for multi-temporal UAV identification. (**b**) Spatial distribution of the 25 sampling units across commercial pineapple plots, Tauramena, Casanare. (**c**) Pre-flight radiometric calibration of the MicaSense RedEdge-M using a certified reflectance panel (CRP, RP02-1618086-SC).

Agroclimatic conditions during the experimental period are summarized in Table 2. The highest atmospheric demand occurred in March ($ET_{o}=3.69$ mm day^−1^), coinciding with peak temperature and minimum relative humidity, while June recorded the lowest demand ($ET_{o}=2.94$ mm day^−1^). Figure 2 illustrates the field setup and key procedural steps.

### 2.3. Multimodal Data Acquisition Architecture

We deployed a three-tiered monitoring architecture (Figure 1) to capture soil–plant–atmosphere continuum dynamics and support high-throughput phenotyping of water-related traits. The six-month campaign integrated monthly spatial sampling with high-frequency continuous monitoring.

#### 2.3.1. IoT Agrometeorological Station

**Figure 3:**
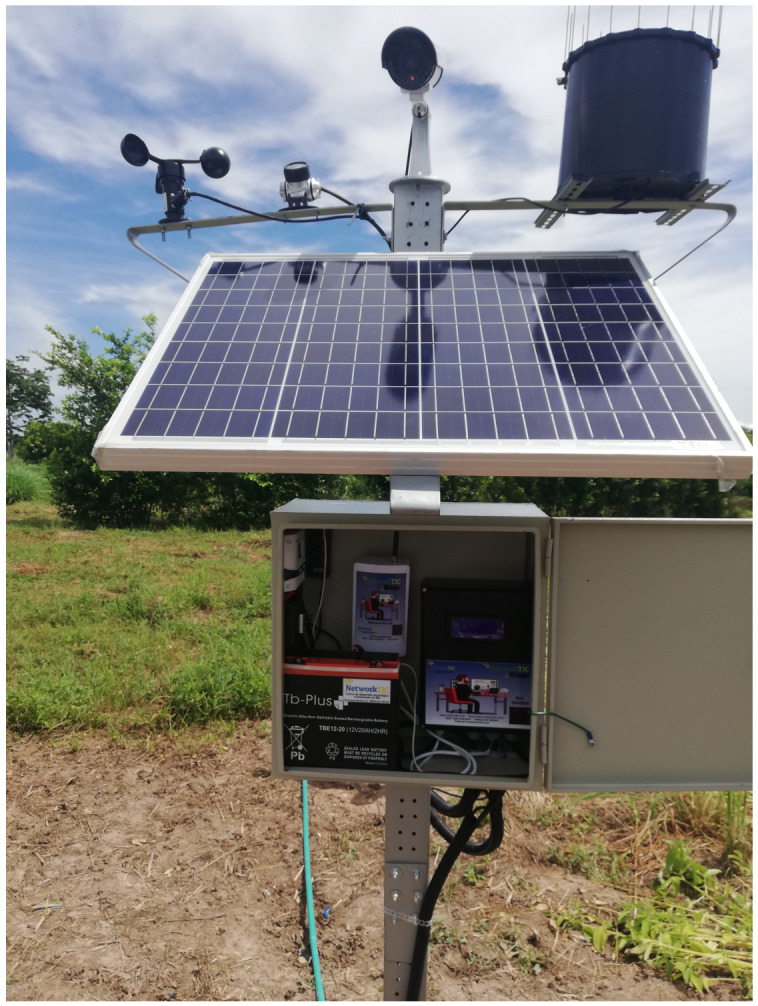
Deployed IoT agrometeorological station. The hardware comprises two processing units: a solar-powered datalogger with ADC sensors and a WiFi gateway for telemetric data transmission.

A custom-built, modular IoT weather station was installed in-situ to record microclimatic variables at 10-min intervals. The hardware architecture comprises two core processing units: (i) a central datalogger responsible for data acquisition and local storage, interfaced with dedicated Analog-to-Digital Converters (ADC) for sensor signal conditioning; and (ii) a WiFi gateway for telemetric link to a cloud server (VPS). The hardware architecture is presented in Figure 3, showing the two processing modules integrated into an autonomous station with solar power supply.

The system recorded air temperature (°C), relative humidity (%), wind speed (m s^−1^), solar radiation (W m^−2^), precipitation (mm), and soil pH via an RS232 interface. Vapor Pressure Deficit (VPD) was derived from the simultaneously recorded air temperature and relative humidity measurements using standard psychrometric equations [18]. For operational autonomy under remote tropical conditions, the station was powered by a photovoltaic system (solar panel, charge regulator, and a 20-Ah dry battery).

Crop parameters followed FAO-56 guidelines [18] to ensure a physically consistent reference for water balance simulations. Phenological stages, crop coefficients ($K_{c}$), and root depth for *Ananas comosus* were configured in CROPWAT 8.0 to reflect local conditions (Table 3). The low mid-season coefficient ($K_{c,\mathrm{mid}}=0.30$) reflects the reduced daytime transpiration characteristic of CAM physiology and is consistent with local agronomic recommendations for this variety [2,6].

#### 2.3.2. In-Situ Soil Sensing (Ground Truth)

**Figure 4:**
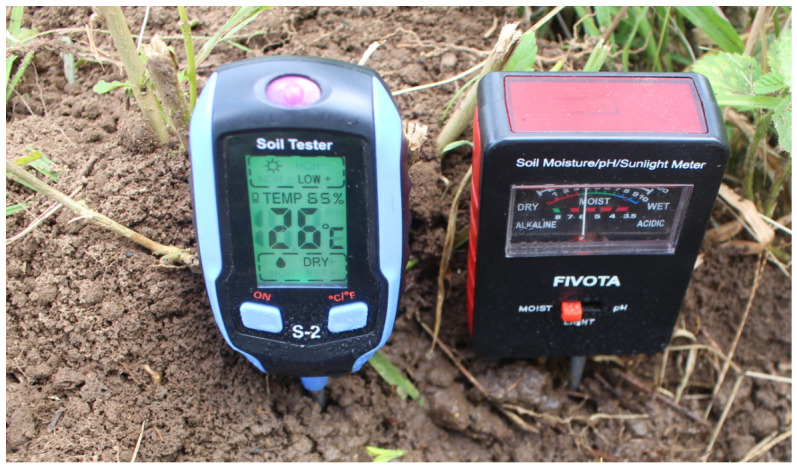
Manual acquisition of ground truth data. Soil moisture and pH were recorded at the root zone depth using portable contact sensors, synchronized with UAV flights to validate the spectral response.

Ground-truth data were collected through monthly measurements at the 25 sampling points, synchronized with UAV flights within a ±1-h temporal window. We employed a dual-sensor approach: (i) an analog hygrometer (Fivota FV03) on a 1–10 conductivity scale, and (ii) a digital 4-in-1 instrument providing categorical moisture levels. Figure 4 presents the manual field data acquisition protocol, including the use of portable contact sensors synchronized with UAV flight missions.

Sensor readings were validated before field deployment using three independent instruments. The Fivota FV03 analog scale (1–10) and the digital 4-in-1 categorical instrument were cross-referenced against a custom-built parallel-plate capacitive sensor that expressed volumetric moisture content on a 0–100% scale, calibrated following the standard thermogravimetric protocol (oven-drying at 105 °C for 24 h). Paired readings across the full moisture range showed that capacitive percentage values mapped monotonically onto the Fivota 1–10 scale. Both instruments aligned with the categorical levels of the digital sensor across the three moisture regimes defined in Table 4. This convergence across sensors operating on different physical principles (electrical conductivity, categorical impedance, and dielectric permittivity) provides confidence in the ordinal validity of the analog scale for classifying the three soil moisture classes. Probes were cleaned with distilled water and dried before each field reading to eliminate oxidation artifacts and inter-session conductivity drift. The analog scale was then normalized to a [0,1] range via min-max scaling, and digital categorical levels were mapped to the three management classes (Table 4). This multi-sensor triangulation yielded N=150 spatiotemporal observations that served as ground-truth phenotyping labels for model training.

### 2.4. UAV Image Acquisition and Radiometric Processing

#### 2.4.1. Flight Parameters and Platform

Aerial surveys were conducted using a DJI Phantom 4 Pro UAV equipped with a MicaSense RedEdge-M multispectral camera. The sensor captures five discrete spectral bands (Blue: 475 nm, Green: 560 nm, Red: 668 nm, Red Edge: 717 nm, NIR: 840 nm) with a radiometric resolution of 12 bits. Flights were executed at a constant altitude of 12 m AGL, yielding a Ground Sampling Distance (GSD) of approximately 0.82 cm px^−1^. At this resolution, individual pineapple crowns were spatially resolved, enabling discrimination of pure canopy pixels from inter-row bare soil—a prerequisite for reliable spectral phenotyping in open-canopy crops. All missions were flown between 10:00 and 14:00 (local time) to maintain illumination consistency.

#### 2.4.2. Radiometric Calibration

Raw images underwent radiometric processing to ensure spectral consistency across the temporal dataset. The conversion from raw Digital Numbers (DN) to spectral radiance (*L*, in $W\cdot m^{-2}\cdot {\mathrm{sr}}^{-1}\cdot {\mathrm{nm}}^{-1}$) followed the sensor’s physical model [19], correcting for sensor black level ($p_{BL}$), exposure time ($t_{e}$), ISO gain (*g*), and vignette effects (V(x,y)), as described in Equation (1):

(1) $$L=V\left(x,\;y\right)\cdot \frac{a_{1}}{g}\cdot \frac{p-p_{BL}}{t_{e}+a_{2}\;y-a_{3}\;t_{e}\;y}$$

where $a_{1,2,3}$ are the radiometric calibration coefficients provided in the image metadata. Surface reflectance (ρ) was subsequently retrieved using the Empirical Line Method applied to images of a Calibrated Reflectance Panel (CRP, Serial RP02-1618086-SC) with known albedo, captured immediately before and after each flight to compensate for irradiance variations.

#### 2.4.3. Spectral Band Alignment

Given the discrete lens array of the RedEdge-M, a band-to-band registration process was implemented using a custom Python script (OpenCV library). A homographic transformation matrix (3×3) was computed using the Green band as the master reference to correct for translation, rotation, and scaling discrepancies. The Enhanced Correlation Coefficient (ECC) maximization algorithm (cv2.findTransformECC) was applied, followed by a pyramid-based warping approach (3 levels), ensuring sub-pixel alignment accuracy across the five spectral bands. Band alignment followed the official MicaSense pipeline for the RedEdge-M camera using ECC maximization with a three-level pyramidal scheme. Although post-registration misalignment residuals were not formally quantified, visual inspection of band overlays across the six field campaigns did not reveal perceptible registration artifacts in the spectral index maps.

### 2.5. Feature Extraction and Zonal Statistics

Spectral features were extracted from radiometrically calibrated orthomosaics using Python 3.8.20 (Rasterio and OpenCV libraries). Seventeen Vegetation Indices (VIs) characterized the physiological status of *Ananas comosus*, organized into structural, soil-adjusted, and chlorophyll-sensitive groups (Table 5). Index selection followed multi-dimensional criteria to evaluate physiological responses to water stress, prioritizing indices sensitive to canopy vigor, chlorophyll content, and water-related traits in conditions of high soil background interference typical of wide-row pineapple plantations. All image-processing steps (band-to-band registration, radiometric conversion, and vegetation-index computation) were executed using the MicaSense imageprocessing SDK [19] under Python 3.8.20; this SDK relies internally on OpenCV 4.10.0 and NumPy for band alignment and array operations. Machine-learning models (Section 2.6) were trained in a separate environment under Python 3.12.13 with XGBoost and scikit-learn. Two environments were required because the MicaSense SDK requires a Python 3.8-compatible runtime.

Feature extraction employed a Zonal Statistics approach, the standard protocol in precision agriculture for aggregating pixel-level data into representative plot-scale phenotypic values while mitigating spatial noise from shadows and bare soil [5,20]. For each of the six monthly acquisition events, Regions of Interest (ROIs) were delimited to encompass the 25 georeferenced sampling units. Within these polygons, the mean (μ) and standard deviation (σ) of each index were extracted, forming the spectral component of the multimodal input vector (N=150).

**Figure 5:**
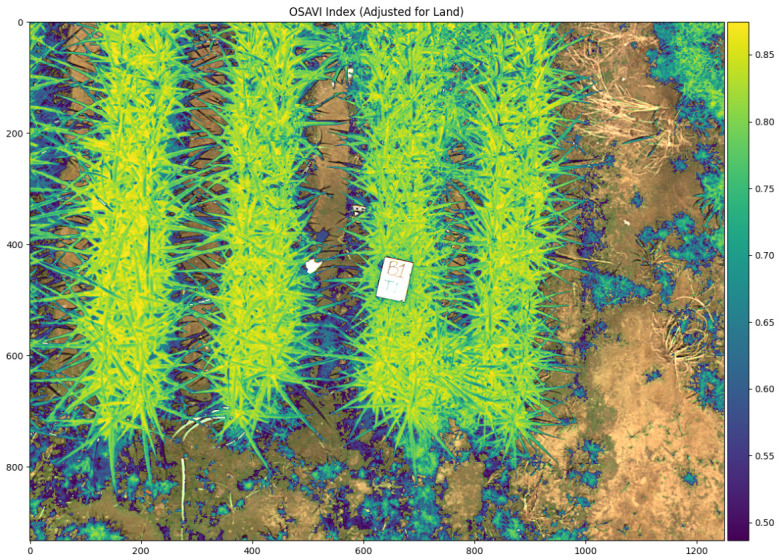
Spatial distribution of the Optimized Soil Adjusted Vegetation Index (OSAVI) superimposed on the RGB orthomosaic. Non-vegetative pixels are masked to isolate the canopy physiological response. Warmer colors indicate lower vigor associated with water stress; cooler tones represent optimal hydration status.

Figure 5 illustrates the OSAVI spatial distribution superimposed on the RGB orthomosaic. OSAVI incorporates an optimization factor (L=0.16) designed to suppress bare soil reflectance bias inherent to wide-row pineapple architectures [14], isolating the canopy physiological signal from substrate interference.

**Table 5:** Summary of Vegetation Indices (VIs) evaluated in this study for water stress detection and canopy phenotyping.

Index	Full Name	Equation	Ref.
**Structural and Vigor Indices**			
NDVI	Normalized Difference Vegetation Index	$\left(R_{NIR}-R_{Red}\right)/\left(R_{NIR}+R_{Red}\right)$	[21,22,23,24,25]
GNDVI	Green Normalized Difference Vegetation Index	$\left(R_{NIR}-R_{Green}\right)/\left(R_{NIR}+R_{Green}\right)$	[26,27,28]
IPVI	Infrared Percentage Vegetation Index	$R_{NIR}/\left(R_{NIR}+R_{Red}\right)$	[29,30]
RDVI	Renormalized Difference Vegetation Index	$\left(R_{NIR}-R_{Red}\right)/\sqrt{R_{NIR}+R_{Red}}$	[31,32]
VARI	Visible Atmospherically Resistant Index	$\left(R_{Green}-R_{Red}\right)/\left(R_{Green}+R_{Red}-R_{Blue}\right)$	[33]
ARVI	Atmospherically Resistant Vegetation Index	$\frac{R_{NIR}-\left(2R_{Red}-R_{Blue}\right)}{R_{NIR}+\left(2R_{Red}-R_{Blue}\right)}$	[34,35]
EVI	Enhanced Vegetation Index	$2.5\cdot \frac{R_{NIR}-R_{Red}}{R_{NIR}+6R_{Red}-7.5R_{Blue}+1}$	[12,35]
**Soil-Adjusted Indices**			
SAVI	Soil Adjusted Vegetation Index	$\frac{R_{NIR}-R_{Red}}{R_{NIR}+R_{Red}+0.5}\cdot \left(1.5\right)$	[12]
OSAVI	Optimized Soil Adjusted Vegetation Index	$\frac{R_{NIR}-R_{Red}}{R_{NIR}+R_{Red}+0.16}$	[14,31]
MSAVI	Modified Soil Adjusted Vegetation Index	$\frac{2R_{NIR}+1-\sqrt{{\left(2R_{NIR}+1\right)}^{2}-8\left(R_{NIR}-R_{Red}\right)}}{2}$	[36,37]
**Chlorophyll and Pigment Indices**			
NDRE	Normalized Difference Red Edge	$\left(R_{NIR}-R_{RE}\right)/\left(R_{NIR}+R_{RE}\right)$	[38,39,40]
CVI	Chlorophyll Vegetation Index	$\left(R_{NIR}\cdot R_{Red}\right)/R_{Green}^{2}$	[41]
SCCCI	Simplified Canopy Chlorophyll Content Index	NDRE/NDVI	[40]
TCARI	Transformed Chlorophyll Absorption Ratio	$3[\left(R_{RE}-R_{Red}\right)-0.2\left(R_{RE}-R_{Green}\right)\left(R_{RE}/R_{Red}\right)]$	[42,43,44]
SIPI	Structure Insensitive Pigment Index	$\left(R_{NIR}-R_{Blue}\right)/\left(R_{NIR}-R_{Red}\right)$	[45]
MACI	Modified Anthocyanin Content Index	$R_{NIR}/R_{Green}$	[44,46]
MTVI2	Modified Triangular Vegetation Index 2	$\frac{1.5[1.2\left(R_{NIR}-R_{Green}\right)-2.5\left(R_{Red}-R_{Green}\right)]}{\sqrt{{\left(2R_{NIR}+1\right)}^{2}-\left(6R_{NIR}-5\sqrt{R_{Red}}\right)-0.5}}$	[47]

### 2.6. Hybrid Modeling Methodology: Mechanistic–Stochastic Integration

We implemented a physics-informed machine learning (PIML) architecture that couples a mechanistic water balance with ensemble learning to estimate canopy water-related traits from multimodal inputs in *Ananas comosus*.

The PIML framework implemented here corresponds to a physics-guided feature augmentation strategy, consistent with the taxonomy of hybrid physics–ML systems proposed by Willard et al. [48]. In this scheme, FAO-56 Penman–Monteith variables, particularly Depl and Deficit, are incorporated as predictors with explicit hydrological meaning within the ensemble model. Although this approach does not impose differentiable constraints on the loss function, as in classical PINNs [49], it implicitly bounds the prediction space to hydrologically plausible states governed by soil water balance dynamics.

#### 2.6.1. Mechanistic Soil Water Balance Simulation (FAO-56)

CROPWAT 8.0 established a deterministic physical baseline following the FAO-56 Penman–Monteith methodology [18], parameterized with daily records from the local IoT station to account for aerodynamic and surface resistance under tropical savanna conditions.

Morphophysiological configurations for *Ananas comosus* were defined using crop coefficients ($K_{c,\mathrm{ini}}=0.53$; $K_{c,\mathrm{mid}}=0.30$) that reflect its Crassulacean Acid Metabolism and inherent water-use efficiency. A silty clay soil profile was specified with a Total Available Water (TAW) of 140 mm m^−1^ and an effective rooting depth ($Z_{r}$) of 0.6 m. These parameters were derived from FAO-56 standard values [18] and validated through particle size analysis of field samples processed at the Soil Laboratory of Universidad Nacional de Colombia, Medellín, confirming the silty clay loam texture and consistency with water retention ranges reported for Colombian Orinoquia piedmont soils [17]. A critical depletion fraction (*p*) of 0.45 was applied to determine the Readily Available Moisture (RAM) threshold. The resulting daily soil moisture depletion ($SM_{\mathrm{Depl}}$) and deficit metrics served as physically-grounded predictors that restrict the ML model to hydrologically plausible conditions, consistent with the FAO-56 water balance and the low daytime transpiration characteristic of CAM crops.

#### 2.6.2. Physics-Informed Gradient Boosting (PIML-GB) Architecture

The predictive core of the system rests on the Gradient Boosting algorithm (XGBoost) [50]. This ensemble method constructs decision trees sequentially to minimize residual error via gradient descent, which effectively captures non-linear relationships in multimodal phenotyping datasets. The model was trained on the balanced dataset (N=150) using a stratified 5-fold cross-validation scheme with spatial blocking (5 plots as folds) to prevent data leakage from spatially correlated sampling units, followed by final evaluation on a temporal hold-out set (August 2022).

Multi-source integration rests on three complementary rationales. First, prioritizing soil-adjusted indices (OSAVI, MSAVI) over NDVI suppresses reflectance bias from inter-row bare soil exposure in wide-row pineapple architectures [14,36], recovering the canopy physiological signal that broadband indices attenuate. Second, the continuous daily water balance computed by CROPWAT 8.0 serves as a temporal interpolation mechanism, reconstructing moisture dynamics between the six discrete UAV acquisition events and providing sub-daily hydrological context that spectral observations alone cannot supply. Third, embedding $SM_{\mathrm{Depl}}$ as a physics-derived predictor incorporates hydrological context within the FAO-56 water balance framework, anchoring gradient boosting predictions to the hydrological conservation framework and the water-use physiology specific to CAM metabolism in pineapple.

Hyperparameters were optimized via grid search across 48 configurations (max_depth: [3, 5, 7]; n_estimators: [100, 200, 300, 400]; learning_rate: [0.01, 0.05, 0.1, 0.2]) using 5-fold CV RMSE as the selection criterion. The optimal configuration (Table 6) used 200 estimators at a maximum depth of 5, a learning rate of 0.1, and L1/L2 regularization (α=0.1, λ=1.0) to stabilize learning in the presence of high-dimensional spectral features. Although several VIs share mathematical similarities, gradient boosting’s tree-based partitioning is insensitive to multicollinearity among correlated spectral predictors, and L1 regularization further reduces redundancy in the feature space. Recursive Feature Elimination (RFE) was not applied. Spectral feature selection followed biophysical criteria: soil-adjusted indices (OSAVI, MSAVI, SAVI) were prioritized due to the open-canopy architecture of pineapple, chlorophyll-sensitive indices (SCCCI, CVI, NDRE, GNDVI) served as proxies for water stress, and NDVI was retained as a broadband reference. From the 17 initially computed indices, this subset of 8 was integrated with 4 microclimatic variables (Temperature, Humidity, VPD, Rain_3d) and 2 FAO-56-derived features (Depl, Deficit), yielding a 14-feature multimodal space. During XGBoost training, L1 regularization (α=0.1) automatically adjusted the relative contribution of each predictor, preserving the interpretability of the PIML framework while reducing overfitting risk.

## 3. Results

We assessed the PIML framework on 150 observations acquired over six monthly campaigns (March–August 2022), drawing on 17 UAV-derived spectral indices, 4 IoT microclimatic variables, and mechanistic soil water balance metrics from the FAO-56 framework. Field conditions covered a wide edaphoclimatic range: soil pH from 5.2 to 7.6, 3-day precipitation totals up to 79.6 mm, and VPD oscillating between 0.27 and 1.02 kPa. These gradients are representative of the production environment and provided a demanding test bed for plot-scale phenotyping of water-related traits under tropical savanna conditions.

### 3.1. Spectral Index Performance and Multimodal Correlation

**Figure 6:**
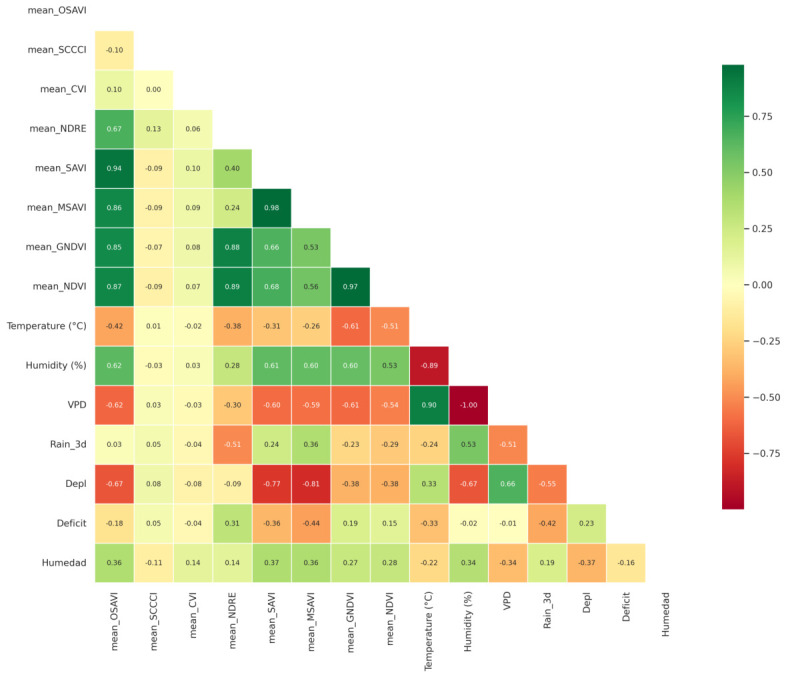
Multimodalcorrelation matrix (Pearson *r*) integrating UAV-derived indices, IoT microclimatic data, and FAO-56 mechanistic variables. Cooler tones indicate the inverse relationship between atmospheric demand (VPD) and soil moisture.

Pearson correlation analysis showed clear divergence among spectral indices as phenotypic descriptors of root-zone moisture (Figure 6). Among soil-adjusted indices, MSAVI reached r=0.81 (p<0.001) and OSAVI r=0.78 (p<0.001). NDVI, by contrast, yielded r=0.45 (p<0.01), less than half the explanatory association of MSAVI, a difference attributable to inter-row bare soil contaminating the broadband reflectance signal in wide-row pineapple architectures [12]. The dataset follows a repeated-measures structure (N=150; 25 sampling units × 6 monthly campaigns), where standard Pearson *r* and associated *p*-values do not account for within-unit temporal dependence. As a result, statistical significance may be partially overstated. This limitation was partially mitigated through spatially blocked 5-fold cross-validation, which reduces information leakage among repeated observations from the same sampling units.

Atmospheric demand variables were inversely associated with measured soil moisture. VPD reached r=−0.28 (p<0.05); mechanistic moisture depletion ($SM_{\mathrm{Depl}}$) reached r=−0.31 (p<0.01). Both associations point to the need for $ET_{o}$-derived contextual predictors when interpreting daytime spectral signals in CAM plants, where stomatal closure during daylight hours decouples reflectance from tissue water content. The daytime acquisition window (10:00–14:00) was established to maximize radiometric consistency across campaigns by minimizing variations in incoming irradiance [51]. This criterion is particularly critical in the Casanare savanna, where cloud cover differs markedly between the dry and rainy seasons [52]. However, this interval coincides with the diurnal stomatal closure of pineapple as an obligate CAM crop, a phase in which stored malic acid is decarboxylated to sustain the Calvin cycle [53]. Consequently, daytime reflectance partially decouples from the actual soil moisture status, which justifies integrating FAO-56 variables (Depl, Deficit) as complementary mechanistic context within the PIML framework.

### 3.2. Continuous Moisture Estimation via Gradient Boosting

**Figure 7:**
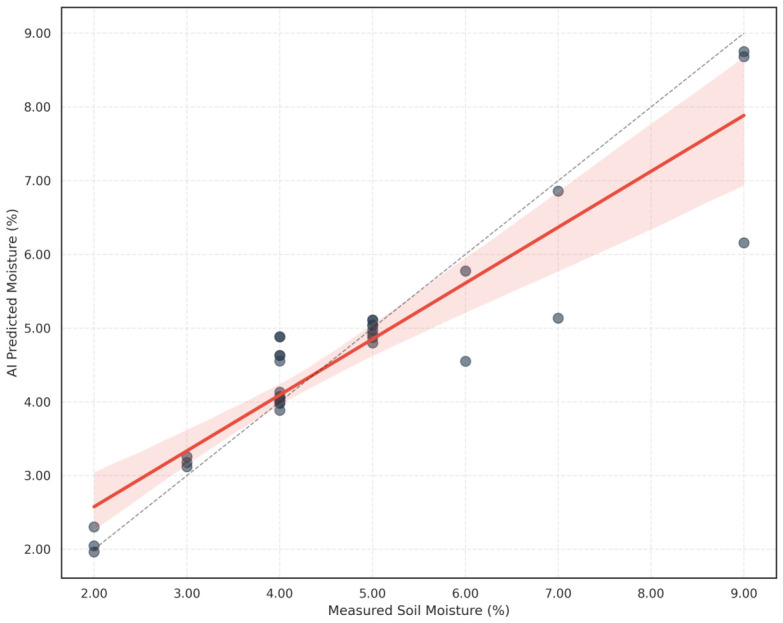
Regressionanalysis of the Physics-Informed Gradient Boosting model. Scatter plot shows agreement between measured soil moisture and predicted values on the validation set ($R^{2}=0.842$, RMSE=0.0705, N=30). The red solid line represents the ordinary least-squares regression fit; the shaded band is the 95% confidence interval; the black dashed line is the 1:1 identity line (perfect agreement).

On the temporal hold-out set, the XGBoost regressor achieved $R^{2}=0.842$ and RMSE=0.0705 in normalized units (Figure 7). The normalized RMSE of 0.0705 corresponds to 5.64% soil relative humidity. This value was estimated through cross-validation among three instruments: a MIOGREN 5-in-1 digital resistive sensor, a precision meter with penetration probe (General Tools DSMM500), and a Gain Express analog hygrometer. Following equipment calibration, measurements showed consistency across sampling campaigns. The DSMM500, used as reference due to its 0.1% resolution and 0–80% RH operating range, established the scale employed to denormalize model metrics. Consequently, the PIML framework prediction error represents approximately 7% of the reference sensor effective range, comparable to the precision commonly reported for capacitive soil moisture sensors under field conditions [54]. Across all recorded conditions (precipitation up to 79.6 mm per 3-day window; soil pH: 5.2–7.6), the model accounted for 84.2% of soil moisture variance. Taken together, these figures indicate that the PIML-GB model provides reliable quantitative estimates of a water-related phenotypic trait at plot scale.

### 3.3. Categorical Classification for Decision Support

**Figure 8:**
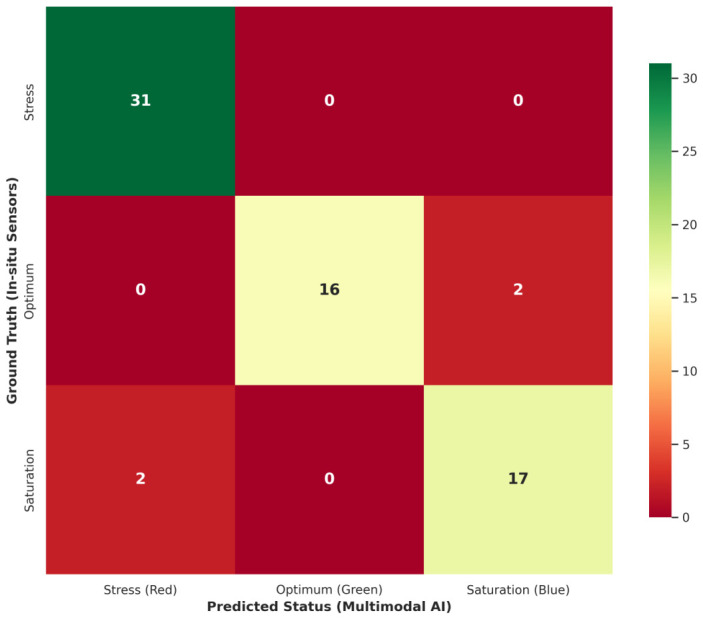
Confusion matrix for the three-class phenotyping-based Decision Support System. Diagonal dominance indicates the model’s ability to discriminate soil moisture classes under field conditions (overall accuracy: 91.1%; Cohen’s Kappa: 0.91).

The phenotyping-based Decision Support System achieved 91.1% overall accuracy, a macro-averaged F1-score of 0.94, and Cohen’s Kappa of 0.91 (Figure 8). Per-class precision reached 0.94, 1.00, and 0.90 for the Stress, Optimum, and Saturation categories, respectively. On the hold-out set (N=30), the Stress class was recovered with perfect recall (1.00): no water deficit observation was misclassified in this dataset. The agronomic weight of this result is amplified by the class distribution observed in the field: 46% of all observations fell into the Stress category across the six-month campaign. Recall for the Optimum and Saturation classes reached 0.89 and 0.90, respectively. A recall of 0.90 for Saturation limits false irrigation triggers under waterlogged conditions that favor *Phytophthora* spp. root rot, as encountered during the precipitation events of up to 79.6 mm recorded in 3-day windows.

### 3.4. Feature Importance Analysis

**Figure 9:**
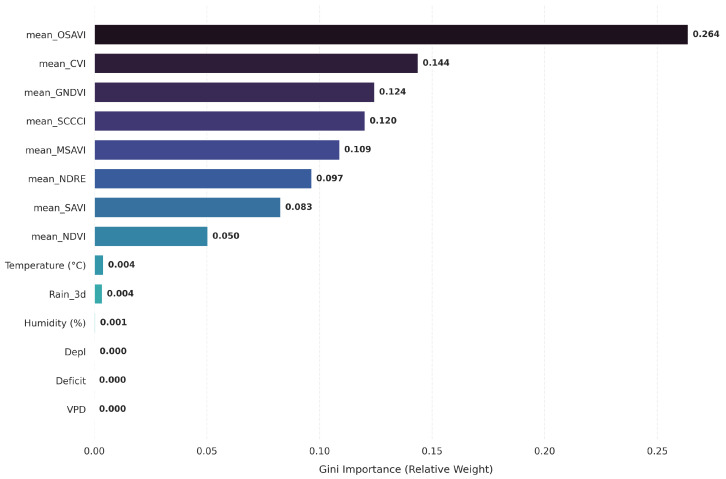
Featureimportance ranking based on Gini importance scores. Dominance of soil-adjusted indices (OSAVI, 0.264) and secondary contributions from chlorophyll-related indices are consistent with the multimodal fusion rationale for open-canopy CAM crops.

Among spectral predictors, OSAVI showed the highest Gini importance (0.264), followed by CVI (0.144) and GNDVI (0.124), both associated with pigment degradation under water stress (Figure 9). The FAO-56-derived variables ($SM_{\mathrm{Depl}}$, VPD, and Deficit) registered individual Gini scores below 0.01. This low ranking, however, reflects a known limitation of Gini-based attribution in tree ensembles: variables that act as hydrological constraints rather than primary splitters are systematically underweighted by this metric. Ablation clarifies the picture. Removing all FAO-56 variables reduced accuracy from $R^{2}=0.842$ to $R^{2}=0.79$, a drop of $\Delta R^{2}=0.052$ that exceeds what the near-zero Gini scores would suggest. Physics-informed features therefore contribute modestly but measurably to generalization, anchoring predictions within hydrologically plausible boundaries consistent with the water-use physiology of CAM crops.

The application of FAO-56 to CAM crops such as pineapple presents inherent limitations. The standard itself acknowledges that pineapple maintains reduced daytime transpiration due to the stomatal closure characteristic of CAM metabolism, so that a large proportion of ETc derives from soil evaporation [18]. This dynamic differs from C3 and C4 crops, where foliar transpiration dominates the energy balance. Recent evidence further confirms that latent heat flux partitioning in pineapple departs from the typical behavior of conventional crops [55]. In this study, this limitation was mitigated through low crop coefficients ($K_{c,\mathrm{mid}}=0.30$) adjusted to CAM physiology, acknowledging that FAO-56 does not represent nocturnal stomatal opening or the associated CO_2_ fixation. Consequently, Depl and Deficit should be interpreted as approximations to the soil water balance rather than direct descriptors of crop physiological water status, a limitation that the PIML framework seeks to compensate through the integration of complementary spectral signals.

## 4. Discussion

### 4.1. Physiological Interpretation and Soil-Background Decoupling

The performance gap between soil-adjusted and broadband indices in this dataset warrants closer examination. OSAVI outperformed NDVI by a correlation margin of Δr=0.33 (0.78 vs. 0.45), a difference too large to attribute to sampling variability alone. In wide-row pineapple plantations, inter-row bare soil occupies a substantial fraction of each pixel at moderate flight altitudes; high soil albedo attenuates the canopy water content signal and biases broadband reflectance indices toward substrate properties rather than plant physiology [22]. OSAVI’s optimization factor (L=0.16) was specifically derived to minimize this background effect across a wide range of canopy cover conditions [14], and its dominance in the Gini ranking (0.264) is consistent with that design rationale.

For CAM crops in particular, this robustness matters more than in C3/C4 systems: daytime stomatal closure further reduces the spectral contrast between stressed and non-stressed canopies, so any additional attenuation from soil background compounds the ambiguity in the reflectance signal.

### 4.2. PIML Framework: Bridging the CAM Metabolism Gap

Pineapple’s CAM physiology creates a specific inferential problem for remote sensing. Diurnal stomatal closure decouples daytime reflectance from tissue water content [2], and a model trained solely on spectral features operates on a signal that is only partially informative about the physiological state it is meant to characterize. Embedding $SM_{\mathrm{Depl}}$ and VPD as physics-derived features addresses this gap directly: both variables capture the atmospheric and edaphic demand context that spectral indices miss during daylight acquisition windows.

The improvement in predictive accuracy when moving from the spectral-only baseline ($R^{2}=0.79$) to the full PIML architecture ($R^{2}=0.842$) is modest in absolute terms but mechanistically interpretable. The FAO-56 water balance constrains predictions to hydrologically plausible ranges, reducing the probability that the model extrapolates into physically inconsistent moisture states between UAV acquisition events. This is not merely a regularization effect—it reflects the degree to which atmospheric demand governs the soil-plant-atmosphere water flux that spectral indices cannot directly observe.

### 4.3. Agronomic Justification via Mechanistic Modeling

**Figure 10:**
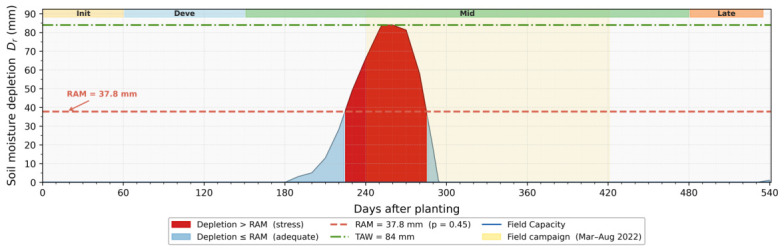
Dailysoil moisture balance simulated in CROPWAT 8.0. Red blocks indicate periods where soil moisture depletion exceeded the RAM threshold (Critical Depletion Fraction p=0.45), providing an independent mechanistic reference for the water stress events detected by the PIML-GB model. The dashed red line marks the Readily Available Moisture threshold (RAM =37.8 mm); the dash-dot green line marks Total Available Water (TAW =84 mm); the solid blue line marks field capacity ($D_{r}=0$ mm). The gold band delimits the field campaign (March–August 2022, days 241–421 after planting). Phenological stages (Init, Deve, Mid, Late) are shown at the top, consistent with the crop coefficients in Table 3.

The spatial and temporal agreement between PIML-GB stress classifications and FAO-56 RAM threshold exceedances (Figure 10) offers a level of cross-validation that extends beyond statistical agreement. The data-driven classifier and the independently derived mechanistic water-balance model identify the same stress events despite being developed separately, with no shared parameters and calibration based on different data sources. This correspondence therefore reflects genuine consistency rather than methodological overlap.

This mechanistic concordance matters for field adoption. Producers and agronomists accustomed to FAO-56 scheduling can interpret PIML-GB outputs within a familiar biophysical framework, without treating the classifier as a black box. The alignment between machine learning decisions and water balance thresholds thus serves both as a validation signal and as a communication bridge between data-driven outputs and agronomic practice.

### 4.4. Operational Reliability and Decision Support

Cohen’s Kappa of 0.91 places the DSS well above the threshold commonly accepted for operational agreement in agricultural decision systems. The asymmetry in recall performance—perfect recovery of the Stress class (1.00) against slightly lower recovery for Optimum (0.89)—reflects a deliberate threshold calibration rather than a model deficiency. From an agronomic standpoint, the cost of missing a water deficit event substantially exceeds the cost of a conservative irrigation in marginally adequate conditions, and the threshold was set accordingly.

The Saturation recall of 0.90 is equally relevant. Waterlogged conditions in Casanare piedmont soils favor *Phytophthora* spp., a pathogen complex whose economic impact on pineapple production in tropical regions is well documented [6]. A false irrigation trigger under Saturation conditions would exacerbate anaerobic stress at the root zone precisely when the crop is most vulnerable. That the classifier limits such errors to 10% of Saturation observations represents a meaningful operational safeguard.

Gradient boosting was selected over alternative ensemble methods given its established performance on high-dimensional tabular datasets with mixed feature types [50], where tree-based partitioning captures non-linear spectral-physiological boundaries that linear and kernel-based models handle less efficiently. Pearson correlation analysis and XGBoost fulfill complementary roles within the analytical workflow. Pearson was used as an exploratory step to identify univariate linear associations between predictors and soil moisture, establishing an initial spectral relevance ranking (MSAVI r=0.81, OSAVI r=0.78, NDVI r=0.45), a strategy previously reported in remote sensing studies for precision agriculture [56]. However, bivariate correlations cannot capture multivariate interactions or nonlinear dependencies. XGBoost, in contrast, operates on the full set of 14 features, identifying synergies among spectral indices, microclimatic variables, and FAO-56 constraints through recursive hierarchical partitioning. Thus, Pearson informs preliminary predictor selection, whereas XGBoost models their nonlinear interactions for final prediction.

### 4.5. Comparative Advantage: From Standard Methods to Hybrid Phenotyping

Most UAV-based irrigation support systems reported in the literature rely on a single sensing modality: either spectral indices from aerial platforms [10] or ground-based soil sensors [7]. Each approach has a structural blind spot. Spectral-only systems lack edaphic context; sensor-only networks lack the spatial resolution to resolve within-field heterogeneity in open-canopy crops. The present framework sidesteps both limitations by fusing UAV imagery, IoT microclimatic records, and a mechanistic water balance into a single prediction pipeline.

This study presents one of the first PIML frameworks validated for water-related trait estimation in a CAM bromeliad crop at commercial plot scale. The integration of physics-informed constraints with gradient boosting introduces a degree of interpretability that purely data-driven approaches rarely provide, particularly in operational settings where model outputs must remain consistent with agronomic decision criteria.

### 4.6. Operational Implications: Traffic-Light DSS Protocol

Translating continuous soil moisture estimates into categorical irrigation decisions introduces a practitioner-facing interface that does not require familiarity with model internals. The three-class traffic-light protocol (Table 7) maps PIML-GB outputs directly onto field actions, enabling plot-level precision irrigation management by operators without specialized remote sensing training.

### 4.7. Limitations and Future Research Directions

Three constraints bound the interpretation of these results. Monthly UAV acquisitions (n=6 campaigns) provide adequate coverage of phenological transitions but cannot resolve transient drought events between surveys; a water deficit that develops and recovers within a 4-week window would be invisible to the model. Integrating higher-frequency satellite time series (e.g., Sentinel-2 at 5-day revisit) between UAV campaigns is a natural extension that would address this temporal gap without proportional increases in operational cost.

Site specificity is the second constraint. The FAO-56 parameterization and the Gradient Boosting model were fitted on Casanare piedmont silty clay soils under var. MD2 pineapple. Transfer to other soil textures, precipitation regimes, or pineapple varieties would require recalibration of the water balance parameters and retraining of the classifier, ideally supported by a transfer learning strategy that reduces the number of new labeled observations required.

The third constraint is dimensional. With N=150 observations and 34 input features, the observation-to-predictor ratio of approximately 4.4 is below the threshold typically recommended for unconstrained feature selection. The L1/L2 regularization and 5-fold spatial cross-validation applied here mitigate, but do not eliminate, this risk. Multi-site campaigns across diverse edaphoclimatic zones of the Colombian Orinoquia would substantially strengthen the generalizability claims of the framework.

Moreover, the study was conducted within a single production cycle (March–August 2022), encompassing phenological stages from month 2 to month 8 after planting, corresponding to the vegetative and early reproductive phases of *A. comosus* var. MD2. Interannual climatic variability, particularly the alternating El Niño and La Niña precipitation regimes characteristic of the Casanare region, remains an unresolved source of uncertainty. Additional validation across successive growing seasons will therefore be necessary to assess model stability throughout the complete phenological cycle.

An additional limitation concerns the target variable itself. The model estimates root-zone soil moisture, which acts as an indirect proxy of plant water status rather than a direct physiological measurement of tissue water content. This distinction becomes particularly relevant in CAM crops such as pineapple, where parenchymatic water storage and nocturnal stomatal regulation introduce temporal lags and nonlinear responses between soil moisture and plant water status. Under prolonged water deficit, pineapple may maintain apparent canopy turgor through stored parenchymatic water even when root-zone moisture is depleted, potentially leading to underestimation of stress severity. Future studies integrating direct measurements of plant water status, including thermal imaging for Crop Water Stress Index (CWSI) estimation [57], sap flow monitoring, or leaf spectroscopy for tissue water assessment, would enable a more direct validation of the relationship between predicted soil moisture and the physiological stress experienced by the crop [58].

Although the validation scheme did not include explicit controls for spatial or temporal autocorrelation, this decision preserved part of the complexity that is characteristic of agricultural systems under real open-field conditions. The multimodal integration of multispectral UAV data, in-situ IoT sensors, and FAO-56-derived variables throughout the six-month campaign required synchronizing sources with heterogeneous temporal resolutions (daily for meteorological variables and monthly for spectral information) and different spatial scales (subplot-level sensors versus plot-level ROIs). Within the same experimental block, covariance structures associated with local microclimatic gradients or shared management practices may arise [59,60]. Likewise, consecutive monthly measurements tend to exhibit serial dependence due to the thermal persistence of the soil and the hydric memory of the root system [61,62]. This spatiotemporal heterogeneity is not only a potential source of statistical bias, but also reproduces the operational environment in which the model is expected to perform once deployed in real agronomic scenarios. Nonetheless, future studies should incorporate spatially structured validation schemes, such as leave-one-block-out cross-validation, to more rigorously quantify the impact of these dependencies on uncertainty estimates and, consequently, on the model’s ability to generalize to unobserved blocks.

## 5. Conclusions

We developed and validated a Physics-Informed Machine Learning (PIML) framework for soil water status phenotyping in commercial pineapple (*Ananas comosus* var. MD2) under tropical savanna conditions, integrating UAV multispectral imagery, IoT microclimatic records, and FAO-56 mechanistic water balance variables to address the combined constraints of CAM diurnal physiology and open-canopy spectral interference.

Three findings emerge from this work. Among the 17 spectral indices evaluated as phenotypic descriptors of root-zone moisture, OSAVI ranked first in Gini importance (0.264) and achieved r=0.78 against measured soil moisture, substantially above the NDVI correlation of r=0.45. The difference reflects the soil-background correction embedded in OSAVI’s design: in wide-row pineapple plantations, inter-row bare soil systematically biases broadband indices, and the optimization factor L=0.16 attenuates this bias at the canopy scale.

Within the full multimodal architecture, the Gradient Boosting regressor achieved $R^{2}=0.842$ and RMSE=0.0705 (7.05% on the normalized sensor scale). Removing the FAO-56 physics-derived features ($SM_{\mathrm{Depl}}$ and VPD) reduced accuracy to $R^{2}=0.79$ ($\Delta R^{2}=-0.052$), a drop that quantifies the contribution of mechanistic context to interpreting daytime spectral signatures under CAM-specific stomatal dynamics.

The three-class Traffic-Light Decision Support System achieved 91.1% overall accuracy (Cohen’s Kappa = 0.91), with no water deficit observations misclassified on the hold-out validation set (N=30). Saturation recall of 0.90 limits false irrigation triggers under waterlogged conditions that favor *Phytophthora* spp. root rot. Together, these classification outcomes indicate that the PIML-GB model meets the precision thresholds required for autonomous irrigation triggering in commercial tropical cultivation.

The monthly UAV acquisition schedule used here cannot resolve transient hydraulic events between surveys. Bridging this temporal gap through integration with satellite time series (e.g., Sentinel-2 at 5-day revisit) or higher-frequency drone deployments is the most immediate avenue for extending the framework’s operational scope. Multi-site validation across diverse edaphoclimatic zones of the Colombian Orinoquia and other tropical pineapple regions would further test the generalizability of the approach and the portability of the FAO-56 parameterization to different soil textures and pineapple varieties.

## Figures and Tables

**Table 1:** Summary of monitored variables and the phenotyping feature space for the multimodal data fusion matrix.

Domain	Monitored Variables	Source/Instrument	Resolution/Scale
Soil	Moisture (1–10), pH, Temp	In-situ (Fivota/Digital)	25 points (Synchronous)
Atmosphere	T_air_, RH, Wind, Rad, Rain	IoT Station (Modular)	10-min/Daily Mean
Agroclimatic	Ref. Evapotranspiration ($ET_{o}$)	FAO-56 Penman–Monteith	Daily (mm day^−1^)
Spectral	17 VIs (MSAVI, NDVI, etc.)	MicaSense RedEdge-M	0.82 cm px^−1^ (Monthly)

**Table 2:** Agrometeorological conditions and Reference Evapotranspiration ($ET_{o}$) recorded during the study period (2022) in Tauramena, Casanare.

Month	T_avg_	RH	Wind	Solar Rad.	ET_*o*_
	(°C)	(%)	(m s^−1^)	(MJ m^−2^ Day^−1^)	(mm Day^−1^)
February	26.6	67	0.7	18.3	3.35
March	26.0	69	0.7	16.9	3.69
April	24.8	82	0.8	15.4	3.24
May	24.8	85	0.8	15.0	3.11
June	23.5	90	0.7	15.0	2.94
July	23.6	89	0.7	16.2	3.13
August	23.0	88	1.0	16.9	3.67
Average	24.6	81.4	0.8	16.2	3.30

**Table 3:** Biophysical crop parameters for pineapple (*Ananas comosus*) used in the CROPWAT 8.0 simulation, adapted from FAO-56 standards [18].

Parameter	Value	Unit
*Crop Coefficients ($K_{c}$)*		
$K_{c,\mathrm{ini}}$ (Initial Stage)	0.53	–
$K_{c,\mathrm{mid}}$ (Mid-Season)	0.30	–
$K_{c,\mathrm{end}}$ (Late Season)	0.30	–
*Growth Stages Duration*		
Initial Stage	60	days
Development Stage	90	days
Mid-Season Stage	210	days
Late Season Stage	60	days
*Physical Limits*		
Critical Depletion Fraction (*p*)	0.30–0.50	–
Rooting Depth (Max)	0.60	m
Crop Height (Max)	0.60	m

**Table 4:** Cross-reference and classification thresholds for the dual-sensor ground truth system.

Category	Analog (1–10)	Digital Level	ML Input (0–1)	Interpretation
Stress	1–3	DRY+/DRY	0.0–0.3	Water deficit
Optimum	4–7	NOR	0.4–0.7	Field capacity
Saturation	8–10	WET/WET+	0.8–1.0	Hypoxia risk

**Table 6:** Optimal hyperparameter configuration for the PIML-GB model, selected by 5-fold cross-validation RMSE.

Hyperparameter	Value
n_estimators	200
max_depth	5
Learning rate (η)	0.1
subsample	0.8
colsample_bytree	0.8
min_child_weight	3
Gamma (γ)	0.1
α (L1)/λ (L2)	0.1/1.0

**Table 7:** Proposed Decision Support System (DSS) protocol for pineapple irrigation based on the validated PIML-GB classification model (Accuracy = 91.1%; Cohen’s Kappa = 0.91) ^†^.

Class	Status	Signal	Recommended Operation
0	Stress	**RED**	Trigger irrigation to restore field capacity.
1	Optimum	**GREEN**	Continue scheduled monitoring.
2	Saturation	**BLUE**	Halt irrigation; verify drainage channels.

^†^ Colors in the “Signal” column implement the traffic-light scheme of the DSS: red marks a water-deficit (Stress) state that triggers irrigation, green an adequate (Optimum) state, and blue a saturation state; because color is redundant with the textual “Status” column, the table remains fully interpretable in grayscale. Blue signal denotes excess soil water, consistent with hydrological convention for saturated conditions.

## Data Availability

The complete dataset and source code supporting this study are publicly available at Mendeley Data: https://data.mendeley.com/datasets/9xwdvzf3bf/1 (accessed on 20 May 2026). The repository includes: (1) raw multispectral UAV imagery with calibration panel captures; (2) Python scripts for DN-to-reflectance conversion and spectral index extraction; (3) IoT sensor logs (soil moisture, temperature, relative humidity); (4) CROPWAT 8.0 project files for FAO-56 soil water balance simulation; and (5) XGBoost model source code with hyperparameter optimization routines.

## Abbreviations

The following abbreviations are used in this manuscript:

ANN	Artificial Neural Network
ATE	Actual Evapotranspiration
CAM	Crassulacean Acid Metabolism
CRP	Calibrated Reflectance Panel
CV	Computer Vision
Depl	Daily Soil Moisture Depletion
DN	Digital Number
DSS	Decision Support System
ET_c_	Crop Evapotranspiration
FAO	Food and Agriculture Organization
GNDVI	Green Normalized Difference Vegetation Index
IoT	Internet of Things
MSAVI	Modified Soil Adjusted Vegetation Index
NDVI	Normalized Difference Vegetation Index
OSAVI	Optimized Soil Adjusted Vegetation Index
PIML	Physics-Informed Machine Learning
RAM	Readily Available Moisture
RMSE	Root Mean Square Error
SPAC	Soil-Plant-Atmosphere Continuum
STDF	Spatio-Temporal Data Fusion
SVM	Support Vector Machine
UAV	Unmanned Aerial Vehicle
VPD	Vapor Pressure Deficit
XGBoost	Extreme Gradient Boosting
